# Arthroscopic Primary Repair of Proximal Anterior Cruciate Ligament Tears Using a Continuous Bundle Suture Technique With Simplified Suture Passing

**DOI:** 10.1016/j.eats.2024.103061

**Published:** 2024-06-13

**Authors:** Yizhong Peng, Hong Wang, Wenbo Yang, Chunqing Meng, Wei Huang

**Affiliations:** Department of Orthopaedics, Union Hospital, Tongji Medical College, Huazhong University of Science and Technology, Wuhan, China

## Abstract

Anterior cruciate ligament (ACL) injury is a common knee sports injury, with proximal ACL tears accounting for most cases. Arthroscopic ACL preservation has shown great potential in repairing ACL anatomic and biological function, with less tissue damage and slightly higher failure rates. Although many techniques for repairing the ACL have been developed, there are still many problems with the existing technology, such as the cumbersome operation of the traditional hook and needle breakage of the Scorpion suture passer (Arthrex). Herein, to further improve operational convenience and reliability, we developed a continuous bundle suture technique for primary repair of proximal ACL tears with suture anchor fixation. This technique aims to achieve continuous suturing with no additional auxiliary suture for guiding suturing by using a passer as a pusher in the suture hook to push out the suture loaded in the tip of the hook after the hook passes through the ligament. This technique takes advantage of the economics of the suture hook and the convenience of the Scorpion suture passer, allowing for flexible application of the suture hook to conveniently achieve anteromedial and posterolateral bundle repair for patients with proximal ACL tears.

Anterior cruciate ligament (ACL) injury is a common knee joint sports injury, with an incidence rate of up to 85 per 100,000 among people aged 16 to 39 years.^1^ Patients with an ACL injury exhibit not only knee joint swelling, pain, and limited mobility but also long-term knee joint instability, increasing the risk of secondary meniscus and cartilage damage and knee osteoarthritis.^2^ Therefore, timely and effective medical intervention for patients with an ACL injury has become a common consensus. ACL reconstruction remains the gold standard in the treatment of ACL injuries, particularly for athletes with high-level athletic activity.^3^ With the increasing cases of ACL preservation techniques that repair the avulsed ligament and preserve the proprioceptive function of the ACL, systematic reviews and meta-analyses comparing ACL preservation and reconstruction have shown that ACL preservation shows comparable or better functional outcomes, with similar or slightly higher failure rates.^4^, ^5^, ^6^

To our knowledge, modern techniques for ACL repair have short-term follow-up times, without clear evidence about the efficacy and safety of the procedure.^5^ Standardization of suture augmentation for proximal ACL tears is still lacking.

Herein, we report a continuous bundle suture technique with facile suture passing, using anchor fixation and the suture hook to achieve continuous suturing with no additional auxiliary suture for guiding suturing, which can also be adapted to repair meniscal and cuff tears.

## Surgical Technique

**Fig 1:**
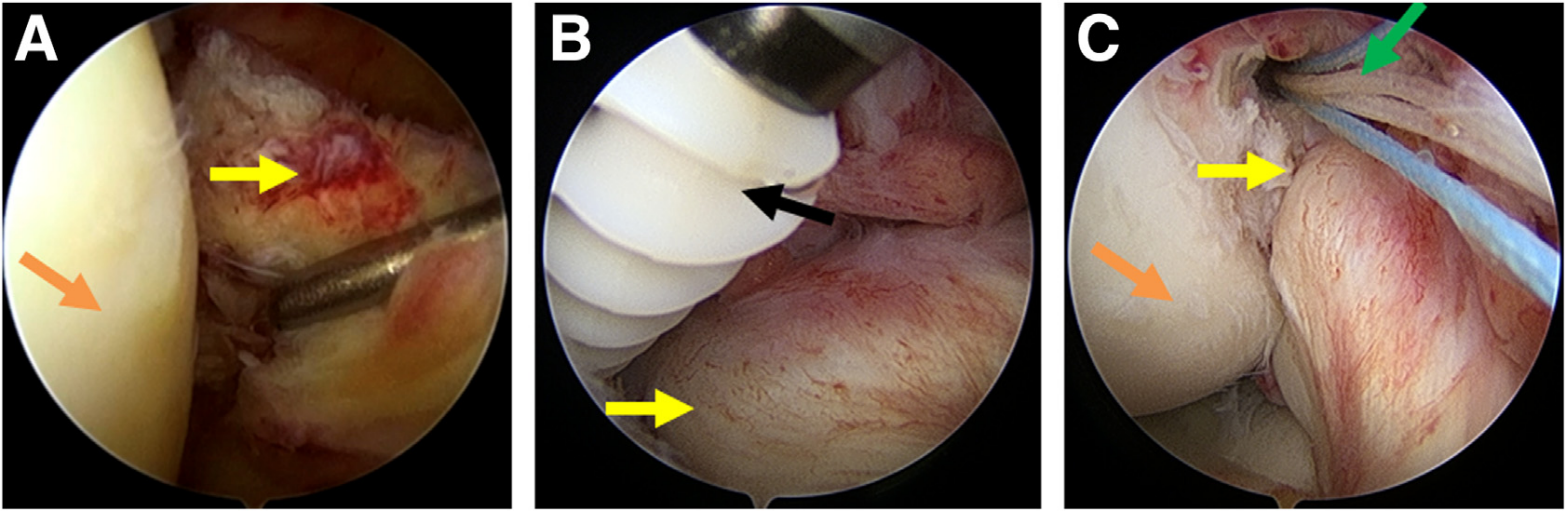
The identification of the proximal anterior cruciate ligament (ACL) tears and placement of an anchor in the right knee. Arthroscopic view from the anterolateral portal (A) to explore the proximal ACL tears and (B) to insert an anchor with double sutures in a footprint of the proximal ACL. (C) Arthroscopic view from the anteromedial portal to observe the inserted anchor with blue and white sutures. Patient was draped in the supine position with 120° knee flexion. Orange arrow: lateral femoral condyle; yellow arrow: femoral insertion point of the ACL; black arrow: anchor; green arrow: anchor sutures.

Our surgical technique is described in detail in Video 1. After general anesthesia, the patient is draped in the supine position, and the affected limb is disinfected in a standardized fashion for arthroscopic ACL surgery. The surgical procedure is recorded through the arthroscopic view in Video 1. Specifically, the acute proximal ACL tear is detected under an anterolateral observation portal (Fig 1A). A 4.5-mm suture anchor (Fastlock PK; Star Sports Medicine) is placed on the footprint of the proximal ACL from the anteromedial portal with 120° knee flexion (Fig 1 B and C).

**Fig 2:**
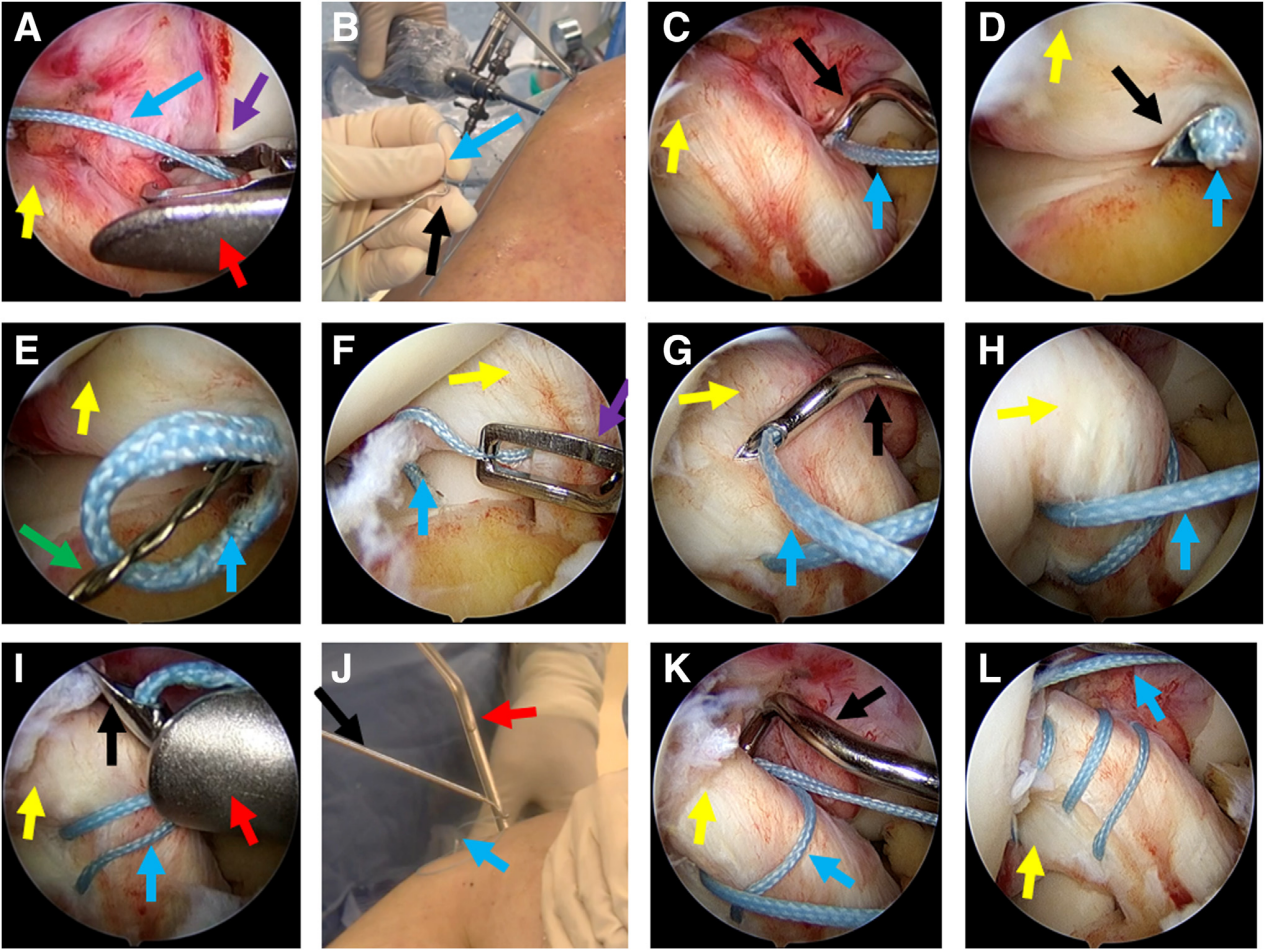
Consecutive suturing processes of the blue anchor suture with the anterolateral portal for observation and the anteromedial portal for operation. (A) The blue anchor suture is grabbed through the anteromedial portal with assistance of the retractor. (B) The tail end of the blue anchor suture is inserted into the tip of the left-curved 45° suture hook at a depth of 2 to 3 cm, while the passer in the hook is withdrawn by 2 to 3 cm. (C) The suture-loaded hook is used to suture the anterior cruciate ligament (ACL) through the anteromedial portal. (D) The suture-loaded hook passes through the ACL. (E) The passer inside the suture hook pushes the suture out of the hook. (F) The hook is withdrawn, while the blue suture is grabbed out through the anteromedial portal. (G, H) A similar operation is performed to continuously suture for the second time. (I, J) Anteromedial bundle repair sutures for the third time. Patient was draped in the supine position with 90° knee flexion. Blue arrow: blue anchor suture; yellow arrow: ACL; purple arrow: thread grabber; red arrow: retractor; green arrow: passer; black arrow: left-curved 45° suture hook.

Three sutures of the anchor are pulled out of the anterolateral portal, leaving 1 blue suture placed in the anteromedial portal. The anterolateral portal is applied as the viewing portal, and a semi-open surgical malleable graft retractor (Mitek Surgical Malleable Graft Retractor with Meniscal Deployment Gun; Depuy Mitek) is placed in the anteromedial portal. A thread grabber is used to grab the single blue anchor suture left through the anteromedial approach (Fig 2A). The retractor is applied to ensure that the suture pulled out from the articular cavity shares the same portal with the suture hook, which can avoid being stuck between the suture hooking and soft tissue (Table 1). The suture hook with a left curve of 45° is loaded with a passer (CHIA PERCPASSER suture passers; Depuy Mitek), while the tip of the passer is at a distance of around 2 to 3 cm from the suture hook outlet. The assistant helps insert the blue suture into the hook at a length of 2 to 3 cm (Fig 2B). If the depth of the anchor suture inserting into the hook is too short, it will easily fall out of the hook. If it is too long, it will be difficult for the passer to completely push the suture out through the hook (Table 1). The surgeon then inserts the hook with the loaded suture and passer into the joint through the anteromedial portal with the aid of the retractor and sutures the stump body of the ACL along the way (Fig 2C). The closed sleeve is not recommended, which would increase the risk of the suture protruding from the hook (Table 1). The direction of sutures should be from the middle of the ligament to the proximal end (Table 1). After the hook passes through the ligament, the passer is pushed out of the hook to bring the suture out of the hook (Fig 2 D and E), and then the hook is withdrawn. Next, the blue suture that passes through the ACL is grabbed through the anterolateral portal to complete 1 suture (Fig 2F). Then, the procedure is repeated twice more for the blue suture to achieve the anteromedial bundle’s repair sutures (Fig 2G-L).

**Table 1:** Pearls and Pitfalls of the Continuous Bundle Suture Technique

Pearls	Pitfalls
A surgical malleable graft retractor guides the hook or assists suture grabbing.	The retractor cannot be replaced by a closed sleeve.
The depth of the suture loaded into the hook should be 2 to 3 cm.	The sutures should be completely free from the hook after passing through the ligament.
A passer pushes out the suture loaded in the hook.	
The observation portal and operation portal can be switched to facilitate continuous sutures on both posterolateral and anteromedial bundles.	
The continuous bundle suture should start from the middle of the ligament toward the proximal end.	

**Fig 3:**
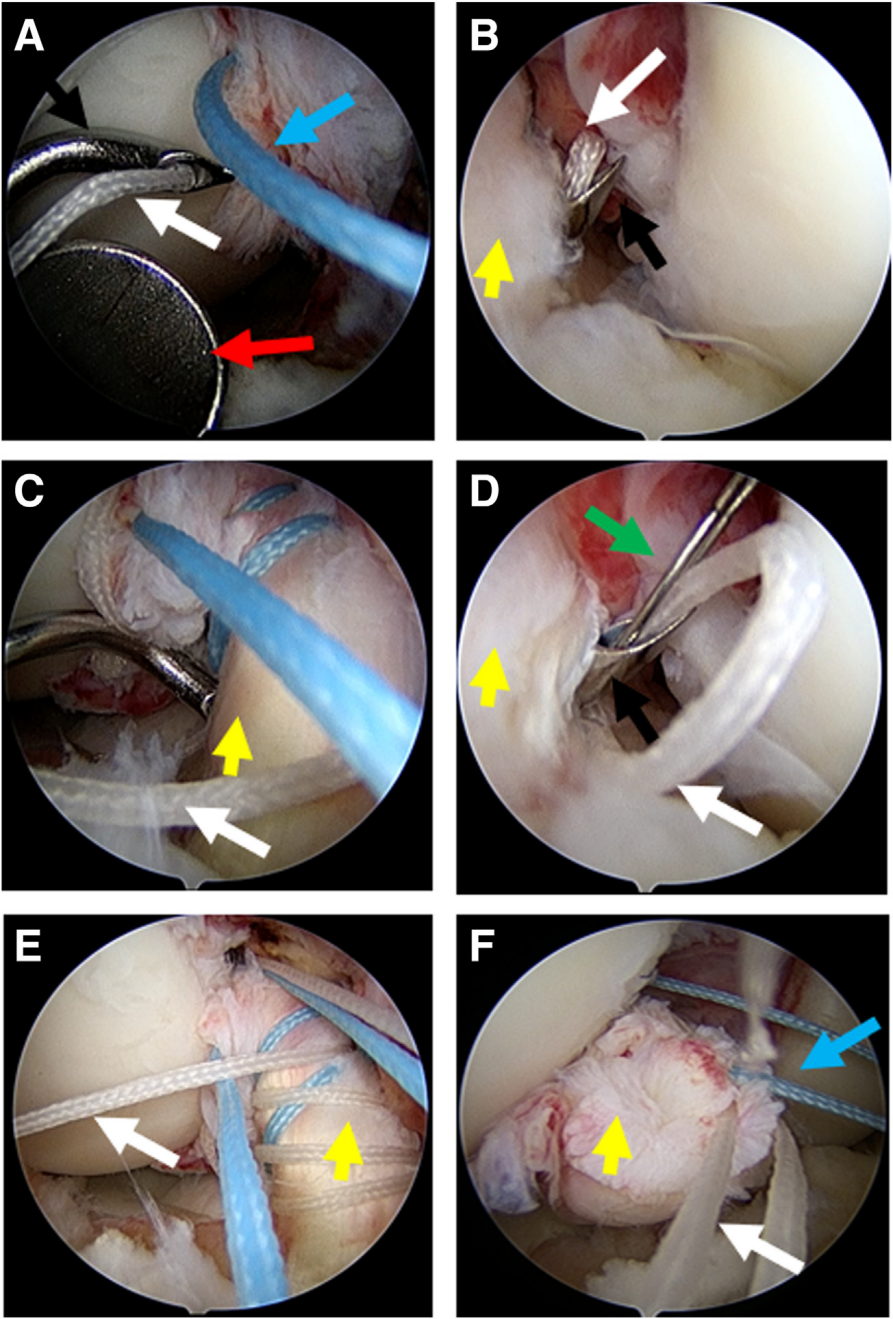
Consecutive suturing processes of the white anchor suture with the anteromedial portal for observation and the anterolateral portal for operation. (A) The right-curved 45° suture hook is applied to load the white suture and inserted through the anterolateral portal with the assistance of the retractor. (B) When the hook passes through the anterior cruciate ligament (ACL), the passer pushes out the white suture. (C) The white suture is grabbed and loaded in the hook to suture the ACL for the second time. (D) The passer pushes out the suture. Arthroscopic view from the anteromedial portal (E) and anterolateral portal (F) after consecutive posterolateral bundle repair sutures for the fourth time. Patient was draped in the supine position with 90° knee flexion. White arrow: white anchor suture; blue arrow: blue anchor suture; yellow arrow: ACL; red arrow: retractor; green arrow: passer; black arrow: right-curved 45° suture hook.

**Fig 4:**
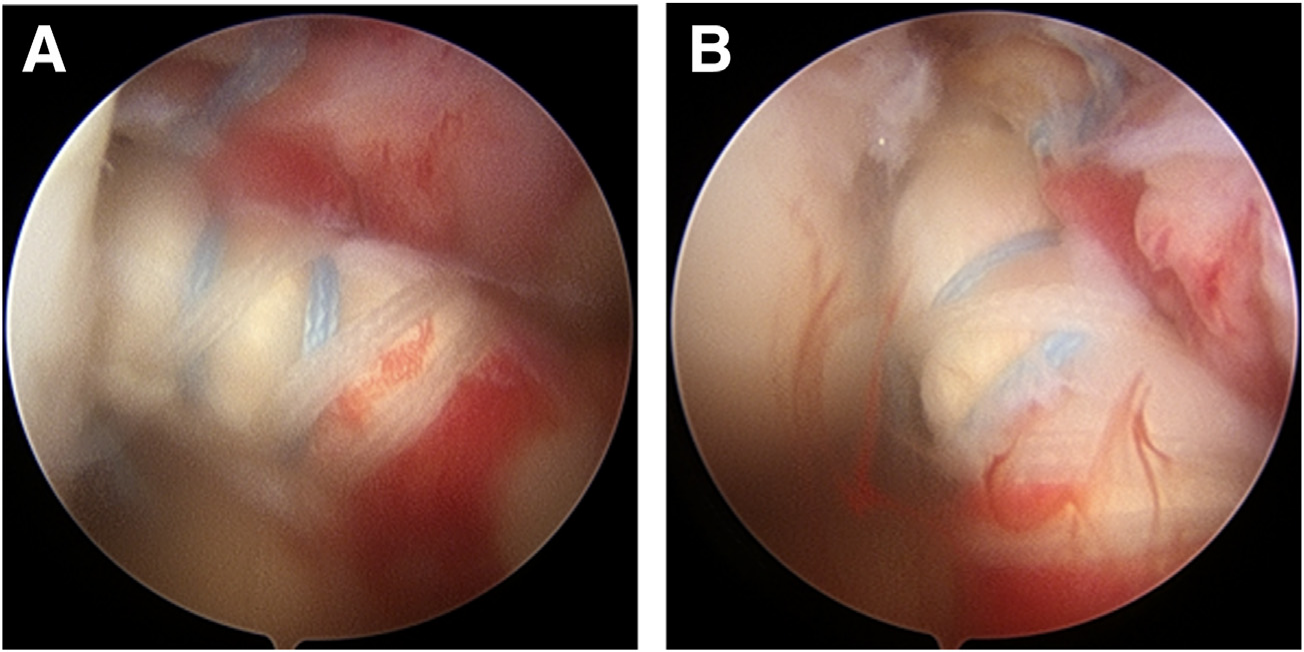
Arthroscopic view from the anteromedial portal (A) and anterolateral portal (B) of the finished anatomic repair of the anterior cruciate ligament. Patient was draped in the supine position with 90° knee flexion.

Then, 1 white suture and 1 blue suture are grabbed from the anterolateral portal to the anteromedial portal, leaving only 1 white suture in the anterolateral portal. Using the anteromedial portal as the observation approach, a similar method is applied to perform 4 consecutive posterolateral bundle repair sutures of the ACL with the white suture and a right-curved suture hook (Fig 3A-F). Through continuous sutures on both anteromedial and posterolateral bundles, the stump of the ACL is sutured in reverse. The number of consecutive sutures for each anchor suture can be adjusted according to the condition of the ligament stump. Then, the anchor sutures are tied and fixed correspondingly to reconstruct the ACL on the femoral footprint area to achieve maximal wall contact (Fig 4).

## Discussion

Because of the importance of rebuilding the function of the ACL, many biologic augmentation techniques have been developed.^7^^,^^8^ ACL reconstruction is mostly performed to address partial and complete ACL tears.^9^ Recently, arthroscopic repair of the ACL has been attracting more attention. ACL repair has the advantage of preserving the native tissue to avoid additional ligament damage of autologous transplantation or the rejection reaction of allogeneic transplantation. Moreover, ACL repair allows for the preservation of proprioceptors and other nerve structures in the ACL, which is important for knee proprioception.^10^

Many techniques for repairing the ACL have been developed, including direct intraligamentary stabilization and fixation using suture anchors, stump sutures, and internal bracing.^11^ Anteromedial and posterolateral bundles have been reported to be a synergistic functional unit to stabilize the knee joint against anteromedial tibial and rotatory forces.^12^^,^^13^ Transitional bundle repairing sutures usually rely on the Scorpion suture passer (Arthrex) or hook to complete the sutures, whereas the hook may result in lower cost and fewer complications.^13^ Traditional suture hooks require the auxiliary suture, such as the PDS thread, to guide the suture passing through the suturing, which is challenging.^14^, ^15^, ^16^

This technique achieves continuous suturing of a single anchor suture and hook without the requirement for traction thread, which saves operating time and increases convenience. Usually, the suture passer, such as the PDS thread, is applied as a guide for suturing after the hook passes sutures through the ligament.^17^ We provided an option for the passer to serve as a pusher to free the suture from the tip of hook. By pushing the passer in the hook, the suture preloaded on the tip of the hook can be conveniently passed through the hook. In this way, the additional auxiliary suture-grabbing approach can be avoided, reducing the efforts of suture management.^18^ This technique, taking the advantage of the suture hook, allows the performance of reverse sutures through different approaches in different directions to conveniently achieve anteromedial and posterolateral bundle repairs,^14^ stabilizing the knee against sports extension forces.^19^^,^^20^ More important, this technique can be widely adapted in other tissue suturing operations, such as meniscus, cuff, and hip joint capsule suturing (Table 2).

**Table 2:** Advantages and Disadvantages of the Continuous Bundle Suture Technique

Advantages	Disadvantages
Continuous suturing with anchor sutures on both anteromedial and posterolateral bundles	Requires familiarity with the application of hook suturing
Less trouble in suture management	The risk of suture detachment when loaded in the hook
Facile suture without auxiliary suture for guiding suturing	
No need for an additional portal to grab suture	
Reverse sutures through different approaches in different directions	
Easy to be adapted to other sutures (e.g., meniscus, cuff, and hip joint capsule tears)	

The disadvantages of this technique should also be stated. For example, suture tangles may occur during continuous suturing. Thus, we recommend the application of a retractor to guide the grabbing of sutures and hook suturing (Table 1). Moreover, familiarity with suture management capabilities should be required. In addition, the suture may fall off when the suture-loaded hook enters the cavity, and when there is no direct fixation between the suture and hook, reloading is inevitable (Table 2).

In this study, we introduce a technique for continuous suturing on anteromedial and posterolateral bundles to achieve anatomic and biomechanical repair of the ACL. By using a passer to push the suture preloaded on the hook tip, this technique has the advantage of both the economics and safety of the suture hook technique and the convenience of the Scorpion technique, aiming to achieve facile repair of proximal ACL tears.

## Disclosures

The authors declare that they have no known competing financial interests or personal relationships that could have appeared to influence the work reported in this paper.

## Supplementary Data

Video 1 Step-by-step surgical technique video of anatomic arthroscopic primary repair for proximal anterior cruciate ligament (ACL) tears. The video demonstrates a proximal ACL tear on a right knee. The anterolateral and anteromedial portals are used as a viewing portal with the patient supine and the knee in 90° of flexion.
